# Association between *MTHFR* C677T polymorphism and cognitive impairment in patients with cerebral small vessel disease: a cross-sectional study

**DOI:** 10.3389/fnagi.2024.1334011

**Published:** 2024-02-19

**Authors:** Qijin Wang, Cuihua Yuan, Zhixiong Zheng, Caihua Chen, Xiao Zhan, Xiaodan Lin

**Affiliations:** ^1^Department of Orthopedics, Affiliated Mindong Hospital, Fujian Medical University, Fuan, China; ^2^Department of Neurology, Affiliated Mindong Hospital, Fujian Medical University, Fuan, China

**Keywords:** cerebral small vessel disease, cognitive impairment, *MTHFR* C677T polymorphism, white matter hyperintensity, hyperhomocysteinemia

## Abstract

**Objective:**

Cerebral small vessel disease (CSVD) is the most common vascular cause of cognitive impairment. This study aimed to explore the association between *MTHFR* C677T polymorphism and cognitive impairment in CSVD patients.

**Methods:**

Demographic, medical, laboratory, cognitive evaluation, and *MTHFR* C677T polymorphism data were collected from CSVD patients admitted to our hospital between January 2019 and July 2023. Inclusion criteria for CSVD were based on the Standards for Reporting Vascular changes on Neuroimaging (STRIVE) criteria, with age ≥ 45 years. Binary logistic regression models were used to analyze risk factors associated with WMH and cognitive impairment.

**Results:**

A total of 330 CSVD participants were recruited in this study, including 179 male and 151 female, with a median age of 64 years (interquartile range: 58–73 years). There were 185 patients (56.1%) with cognitive impairment, 236 patients (71.5%) with WMH, 89 patients (27.0%) with CMB, 87 patients (26.4%) with lacunes. All participants completed *MTHFR* polymorphism analysis, 149 cases (45.2%) of the CC genotype, 112 cases (33.9%) of the CT genotype and 69 cases (20.9%) of the TT genotype. Patients with TT genotype exhibited higher plasma homocysteine levels and more severe WMH and cognitive impairment (*p* < 0.001). Multivariable binary logistic regression model showed that WMH was significantly associated with age (*p* = 0.019), history of hypertension (*p* = 0.011), HHcy (*p* = 0.019) and *MTHFR* genotype (*p* = 0.041); while cognitive impairment was significantly associated with age (*p* = 0.033), history of hypertension (*p* = 0.019), HHcy (*p* = 0.040), *MTHFR* genotype (*p* = 0.039), WMH (*p* = 0.041), and lacunes (*p* = 0.001).

**Conclusion:**

In this cross-sectional study, we investigated the association between *MTHFR* C677T polymorphism and cognitive function in CSVD patients. We found that *MTHFR* 677 TT genotype was an independent risk factor for the progression of WMH and cognitive impairment in CSVD patients.

## Introduction

Cerebral small vessel disease (CSVD) is a chronic progressive cerebrovascular disorder, involving lesions in intracranial small arteries, arterioles, capillaries, and small veins. CSVD is a prominent determinant of cognitive impairment in the elderly, accounting for approximately 50% of dementia cases worldwide (Wardlaw et al., 2019). The clinical manifestations of CSVD demonstrate significant heterogeneity, often lacking apparent clinical symptoms and being under-recognized in early stages. With the emergence and widespread application of neuroimaging techniques, CSVD can be increasingly identified based on its characteristic radiologic features, including white matter hyperintensities (WMH), cerebral microbleeds (CMBs), lacunar infarctions (Wardlaw et al., 2013).

With accelerating aging populations and rising incidence of cerebrovascular risk factors, the prevalence of CSVD continues to rise. However, its pathogenesis remains to be incompletely understood (Wardlaw et al., 2019). In addition to the well-recognized risk factors including blood–brain barrier (BBB) dysfunction, hypoperfusion, oxidative stress, and inflammation, hyperhomocysteinemia (HHcy) has been identified as a potential novel independent risk factor for CSVD (Hassan et al., 2004; Ji et al., 2020). HHcy can promote vascular endothelial damage by oxidative stress mechanisms, accelerate atherosclerotic plaque formation, exacerbate arteriosclerosis, and act synergistically with hypertension to increase the likelihood of stroke (Liu et al., 2017). Methylenetetrahydrofolate reductase (*MTHFR*) is a crucial enzyme in homocysteine metabolism, with over 10 recognized allelic variants. The most common variant is the C677T mutation, which reduces MTHFR enzyme activity and thermostability, constituting a major genetic basis for heightened homocysteine levels.

Although it is possible to identify CSVD manifestations through imaging, these radiological changes often lag behind the onset of lesions. Early detection and intervention before symptoms onset can significantly improve the prognosis of CSVD. Therefore, comprehensive analysis of CSVD risk factors and identification of relevant biomarkers has become particularly critical. Most current studies have focused on the association between CSVD and homocysteine levels, with limited attention to the relationship between *MTHFR* C677T polymorphism and cognitive impairment. Notably, homocysteine levels are susceptible to various external factors, including lifestyle and environmental influences, making it an unstable marker for early detection. Recent studies have indicated that the TT genotype is an independent risk factor for WMH (Li et al., 2022). A meta-analysis of 36 prospective studies demonstrated that WMH is associated with increased risk of cognitive impairment, serving as a neuroimaging marker for dementia (Hu et al., 2021).

Therefore, we collected clinical information and radiological data related to CSVD and assessed cognitive function using the MMSE. The purpose of this study was to explore the association between *MTHFR* C677T polymorphism and cognitive impairment in CSVD patients.

## Materials and methods

### Study design and participants

This single-center, retrospective, cross-sectional study was approved by the ethics committee of our hospital. All participants were recruited from the neurology departments of our institution from January 2019 to July 2023. Written informed consent was obtained from all participants. The sample size was calculated based on the 30.5% value of CSVD prevalence in Chinese population (Yang et al., 2023), with a confidence level of 95% and a margin error of 5%. The sample size required for this study was 330. Inclusion criteria for CSVD were based on the Standards for Reporting Vascular changes on Neuroimaging (STRIVE), with an age of 45 years or older. Exclusion criteria were: (1) history of large cerebral infarction or hemorrhage, (2) white matter lesions secondary to autoimmune diseases, inflammatory conditions, multiple sclerosis, or neoplasms, (3) chronic kidney disease and renal transplantation, (4) abnormal thyroid function, (5) impaired hepatic function or liver diseases, (6) recent use of methotrexate, B vitamins, folic acid, or antiepileptic agents.

### Data collection

We collected demographic and medical information, including age, sex, smoking status, history of hypertension, diabetes mellitus, and hyperlipidemia. Hypertension was defined as self-reported hypertension, treatment with antihypertensive medication, systolic blood pressure ≥ 140 mmHg, or diastolic blood pressure ≥ 90 mmHg. Diabetes mellitus was defined as self-reported diabetes, use of oral antidiabetic drugs or insulin, fasting serum glucose ≥7.0 mmol/L or hemoglobin A1c (HbA1c) ≥6.5%. Hyperlipidemia was defined as fasting serum total cholesterol (TC) >5.2 mmol/L, low density lipoprotein cholesterol (LDL-C) >3.62 mmol/L, or use of lipid-lowering drugs. Smoking status was classified as current smoker (at least within the prior month) or non-current smoker. HHcy was classified as fasting plasma homocysteine ≥15 μmol/L. Venous blood samples, routinely drawn after an overnight fast, were analyzed for plasma TC, LDL-C, glucose, HbA1c and homocysteine.

### *MTHFR* genotyping

Genomic DNA was extracted from peripheral blood leukocytes using a genomic DNA extraction kit (QIAGEN). The *MTHFR* C677T polymorphism was performed by polymerase chain reaction (PCR) with the following primers: forward 5’-TGAAGGAGAAGGTGTCTGCGGGA-3′, reverse 5’-AGGACGGTGCGGTGAGAGTG-3′.

### Neuroimaging data collection

Imaging was performed using a 3.0 T Siemens scanner, included T1-weighted imaging, T2-weighted imaging, diffusion-weighted imaging (DWI), and magnetic resonance angiography (MRA) or computed tomography angiography (CTA). All neuroimaging data were independently evaluated by two experienced Neurologist. WMHs on MRI were visually assessed using the Fazekas scale (0–3): grade 0 = no lesions, grade 1 = focal damage, grade 2 = partial fusion of lesions and grade 3 = diffuse involving the entire area, with or without “U”-fiber involvement. CMBs were defined as homogeneous round or ovoid signal loss on SWI, 2 to 10 mm in diameter with blooming effect. Lacunar infarctions were defined as focal deep infarcts 3 to 15 mm in size, mainly located in basal ganglia or white matter.

### Cognitive function assessment

Cognitive function was assessed using the Mini-Mental State Examination (MMSE). The MMSE has a maximum score of 30 with higher scores denoting better cognitive performance. The MMSE cutoff points for cognitive impairment screening were 17 for illiterate, 20 for individuals with 1–6 years of education, and 24 for individuals with 7 or more years of education (Creavin et al., 2016).

### Statistical analysis

All data were entered into Excel and analyzed using SPSS 26.0 software (IBM, Armonk, New York, United States). Continuous variables were described as means ± standard deviations or medians (interquartile ranges, IQR). Non-normally distributed continuous variables were compared using the Kruskal-Wallis H rank sum test. Categorical variables were expressed as constituent ratios or rates (%), and compared using Pearson chi-squared (*X*^2^) test. The Spearman correlation analysis was employed to assess the association between WMH and cognitive impairment with various factors, accounting for non-normal distribution. Initial univariate binary logistic regression models were performed for each variable with WMHs. Then, multivariable binary logistic regression models were adjusted for age, hypertension, HHcy and *MTHFR* genotype. Finally, univariate and multivariable binary logistic regression analyses were performed for cognitive impairment with age, hypertension, HHcy, *MTHFR* genotype, WMHs, and lacunes as covariates. The results were expressed as odds ratio (OR) and 95% confidence interval (CI). *p* values <0.05 were considered statistically significant.

## Results

### Demographics in CSVD participants

A total of 330 CSVD participants were recruited in this study. The cohort consisted of 179 male and 151 female, with a median age of 64 years (interquartile range: 58–73 years). Among the participants, 154 (46.7%) were current smokers, 183 (55.5%) had a history of hypertension, 100 (30.3%) had diabetes mellitus, 136 (41.2%) had hyperlipidemia, 131 (39.7%) were HHcy, and 185 (56.1%) had cognitive impairment. Participants were categorized into three groups based on neuroimaging characteristics: the WMH group (*n* = 236, 71.5%), CMB group (*n* = 89, 27.0%) and lacunar infarctions group (*n* = 87, 26.4%). The WMH group had a significantly higher proportion of participants compared to the other two groups. Demographic parameters are shown in Table 1.

**Table 1:** Demographic characteristics of CSVD participants.

	Total (*n* = 330)	WMH (*n* = 236)	CMB (*n* = 89)	Lacunes (*n* = 87)
Age, y, median (interquartile range)	64 (58–73)	67 (60–73)	61 (51–69)	67 (59–73)
45–54	50 (47–51)	49.5 (47–51)	50 (48–51)	50 (48–51)
55–64	61 (59–63)	62 (59–63)	60 (59–61)	60 (59–61)
65–74	69 (67–72)	69 (67–72)	69 (66–70)	71 (67–72)
≥75	79 (76–82)	79 (76–84)	77 (76–79)	80 (76–82)
Sex, Male, *n*, (%)	179 (54.2)	131 (55.5)	45 (50.6)	49 (56.3)
Current smoker, *n*, (%)	154 (46.7)	109 (46.2)	42 (47.2)	44 (50.6)
Hypertension, *n*, (%)	183 (55.5)	146 (61.9)	44 (49.4)	46 (52.9)
Diabetes mellitus, *n*, (%)	100 (30.3)	74 (31.4)	33 (37.1)	25 (28.7)
Hyperlipidemia, *n*, (%)	136 (41.2)	98 (41.5)	38 (42.7)	36 (41.4)
TC, mmol/L, median (interquartile range)	4.39 (3.32–5.53)	4.35 (3.34–5.57)	4.42 (3.38–5.42)	4.30 (3.24–5.57)
LDL-C, mmol/L, median (interquartile range)	2.60 (1.65–3.60)	2.62 (1.73–3.59)	2.85 (1.57–3.73)	2.44 (1.52–3.76)
HHcy, (*n*, %)	131 (39.7)	113 (47.9)	31 (34.8)	32 (36.8)
Hcy, μmol/L, median (interquartile range)	11.85 (9.47–17.3)	14.25 (9.90–18.10)	10.6 (8.70–15.55)	11.7 (9.40–17.30)
Cognitive impairment, *n*, (%)	185 (56.1)	151 (64.0)	44 (49.4)	59 (67.8)

WMH, white matter hyperintensity; CMB, cerebral microbleeds; TC, total cholesterol; LDL-C, low density lipoprotein chesterol; Hcy, homocysteine; HHcy, hyperhomocysteinemia.

### Association between *MTHFR* genotype and WMH in CSVD

All participants completed *MTHFR* polymorphism analysis. When categorized by genetic typing, there were 149 cases (45.2%) with the CC genotype, 112 cases (33.9%) with the CT genotype and 69 cases (20.9%) with the TT genotype. Notably, the proportion of TT genotype was lowest among the three genotype groups. Moreover, we confirmed that individuals with the TT genotype exhibited higher plasma homocysteine levels compared to those with the CC and CT genotypes (*p* < 0.001). No statistically differences were observed in terms of age, gender, smoking status, LDL-C, TC, history of hypertension, diabetes and hyperlipidemia. Importantly, there was a statistically significant difference among the three genotype groups in patients with WMH and cognitive impairment (*p* < 0.001), while no statistical difference was found in patients with CMB and lacunes (Table 2).

**Table 2:** Relationships between *MTHFR* genotype and CSVD.

	CC (*n* = 149)	CT (*n* = 112)	TT (*n* = 69)	*p* value
Age, y, median (interquartile range)	63 (58–72)	65 (55–73)	69 (59–74.5)	0.63*
Sex, male, *n*, (%)	80 (53.7)	62 (55.4)	37 (53.6)	0.958^#^
Current smoker, *n*, (%)	73 (49.0)	47 (42.0)	34 (49.3)	0.471^#^
Hypertension, *n*, (%)	77 (51.7)	63 (56.3)	43 (62.3)	0.332^#^
Diabetes mellitus, *n*, (%)	46 (30.9)	34 (30.4)	20 (29.0)	0.961^#^
Hyperlipidemia, *n*, (%)	62 (41.6)	45 (40.2)	29 (42.0)	0.962^#^
TC, mmol/ L, median (interquartile range)	4.41 (3.32–5.69)	4.41 (3.24–5.40)	4.19 (3.44–5.54)	0.891*
LDL-C, mmol /L, median (interquartile range)	2.62 (1.625–3.75)	2.52 (1.625–3.54)	2.63 (1.82–3.59)	0.898*
HHcy, (*n*, %)	34 (22.8)	40 (35.7)	57 (82.6)	<0.001^#^
Hcy, μmol /L, median (interquartile range)	10.1 (8.4–14.5)	13.15 (9.55–16.37)	18.1 (15.55–20.85)	<0.001*
Cognitive impairment, *n*, (%)	65 (43.6)	65 (58.0)	55 (79.7)	<0.001*
**MRI features, n, (%)**				
WMH				
0	58 (38.9)	28 (25.0)	8 (11.6)	<0.001^#^
1	33 (22.1)	43 (38.4)	27 (39.1)	
2	45 (30.2)	19 (17.0)	16 (23.2)	
3	13 (8.7)	22 (19.6)	18 (26.1)	
CMB	46 (30.9)	25 (22.3)	18 (26.1)	0.300^#^
Lacunes	39 (26.2)	27 (24.1)	21 (30.4)	0.642^#^

*_,_ Kruskal-Wallis H rank sum test; ^#^_,_ Pearson chi-squared (*X*^2^) test; WMH, white matter hyperintensity; CMB, cerebral microbleeds; Hcy, homocysteine; HHcy, hyperhomocysteinemia; CC, CC genotype; CT, CT genotype; TT, TT genotype; *p* ≤ 0.05 was considered statistically significant.

### Factors significantly associated with white matter damage

Spearman correlation analysis of relevant risk factors for WMH is shown in Table 3. A positive correlation was observed between the severity of WMH and age (*p* < 0.001), history of hypertension (*p* < 0.001), HHcy (*p* < 0.001), and *MTHFR* genotype (*p* < 0.001).

**Table 3:** Spearman correlation analysis of WMH in CSVD patients.

	ρ	*p* value
Age (y)	0.374	<0.001
Sex	0.068	0.220
Current smoker	0.050	0.365
Hypertension	0.224	<0.001
Diabetes mellitus	0.012	0.832
Hyperlipidemia	0.020	0.723
HHcy	0.305	<0.001
MTHFR genotype	0.193	<0.001

HHcy, hyperhomocysteinemia; *p* ≤ 0.05 was considered statistically significant.

Univariate analysis of binary logistic regression model showed that WMH was significantly associated with age, history of hypertension, HHcy and *MTHFR* genotype. When included in the multivariable model, age (65–74 years, OR: 2.530, 95% CI: 1.213 to 5.279, *p* = 0.013; ≥75 years, OR: 2.860, 95% CI: 1.201 to 6.809, *p* = 0.018), history of hypertension (OR: 1.977, 95% CI: 1.170 to 3.341, *p* = 0.011), HHcy (OR: 2.201, 95% CI: 1.139 to 4.251, *p* = 0.019) and *MTHFR* TT genotype (TT genotype, OR: 2.706, 95% CI: 1.096 to 6.685, *p* = 0.031) remained significant (Table 4).

**Table 4:** Binary logistic regression analysis of risk factors of WMH in CSVD patients.

variables	Univariable analysis		Multivariable analysis	
	OR (95%CI)	*p* value	OR (95%CI)	*p* value
**Age (y)**				
45–54	Ref		Ref	
55–64	1.351 (0.722, 2.528)	0.347	1.522 (0.777, 2.979)	0.221
65–74	3.487 (1.743, 6.977)	<0.001	2.530 (1.213, 5.279)	0.013
≥75	4.185 (1.840, 9.518)	0.001	2.860 (1.201, 6.809)	0.018
*p* for trend		<0.001		0.019
Sex	1.196 (0.741, 1.930)	0.465		
Current smoker	0.935 (0.579, 1.509)	0.782		
Hypertension	2.499 (1.531, 4.080)	<0.001	1.977 (1.170, 3.341)	0.011
Diabetes mellitus	1.195 (0.704, 2.028)	0.510		
Hyperlipidemia	1.047 (0.643, 1.702)	0.855		
HHcy	3.879 (2.185, 6.885)	<0.001	2.201 (1.139, 4.251)	0.019
**MTHFR genotype**				
CC	Ref		Ref	
CT	1.912 (1.115, 3.280)	0.019	1.720 (0.968, 3.056)	0.064
TT	4.860 (2.168, 10.894)	<0.001	2.706 (1.096, 6.685)	0.031
*p* for trend		<0.001		0.041

CI, confidence interval; OR, odds ratio; HHcy, hyperhomocysteinemia; CC, CC genotype; CT, CT genotype; TT, TT genotype; *p* ≤ 0.05 was considered statistically significant.

### Factors significantly associated with cognitive impairment

Spearman correlation analysis of relevant risk factors for cognitive impairment is shown in Table 5. A positive correlation was observed between the cognitive impairment and age (*p* < 0.001), history of hypertension (*p* < 0.001), HHcy (*p* < 0.001), *MTHFR* genotype (*p* < 0.001), WMH (*p* < 0.001) and lacunes (*p* = 0.01).

**Table 5:** Spearman correlation analysis of cognitive impairment in CSVD patients.

	ρ	*p* value
Age (y)	0.335	<0.001
Sex	0.082	0.139
Current smoker	0.045	0.417
Hypertension	0.238	<0.001
Diabetes mellitus	0.066	0.234
Hyperlipidemia	0.059	0.285
HHcy	0.306	<0.001
MTHFR genotype	0.269	<0.001
WMH	0.289	<0.001
CMB	−0.081	0.142
Lacunes	0.142	0.010

WMH, white matter hyperintensity; CMB, cerebral microbleeds; HHcy, hyperhomocysteinemia; *p* ≤ 0.05 was considered statistically significant.

Univariate binary logistic regression analysis showed cognitive impairment was significantly associated with age, hypertension history, HHcy, *MTHFR* genotype, WMH, and lacunes. When included in the multivariable model, age (65–74 years, OR: 2.219, 95% CI: 1.074 to 4.585, *p* = 0.031; ≥75 years, OR: 2.714, 95% CI: 1.185 to 6.214, *p* = 0.018), history of hypertension (OR: 2.009, 95% CI: 1.203 to 3.353, *p* = 0.008), HHcy (OR: 1.874, 95% CI: 1.030 to 3.408, *p* = 0.040), *MTHFR* TT genotype (TT genotype, OR: 2.786, 95% CI: 1.233 to 6.295, *p* = 0.014), WMH (Fazekas grade 2, OR: 2.518, 95% CI: 1.208 to 5.250, *p* = 0.014; Fazekas grade 3, OR: 3.067, 95% CI: 1.225 to 7.680, *p* = 0.017) and lacunes (OR: 2.875, 95% CI: 1.518 to 5.445, *p* = 0.001) remained significant (Table 6).

**Table 6:** Binary logistic regression analysis of risk factors of cognitive impairment in CSVD patients.

Variables	Univariable analysis		Multivariable analysis	
	OR (95%CI)	*p* value	OR (95%CI)	*p* value
**Age (y)**				
45–54	Ref		Ref	
55–64	1.164 (0.621, 2.181)	0.635	1.166 (0.580, 2.342)	0.667
65–74	3.934 (2.062, 7.503)	<0.001	2.219 (1.074, 4.585)	0.031
≥75	4.889 (2.329, 10.261)	<0.001	2.714 (1.185, 6.214)	0.018
*p* for trend		<0.001		0.033
Sex	1.391 (0.898, 2.153)	0.139		
Current smoker	1.199 (0.775, 1.855)	0.415		
Hypertension	2.667 (1.703, 4.176)	<0.001	2.009 (1.203, 3.353)	0.008
Diabetes mellitus	1.337 (0.829, 2.157)	0.234		
Hyperlipidemia	1.274 (0.818, 1.986)	0.284		
HHcy	3.823 (2.357, 6.202)	<0.001	1.874 (1.030, 3.408)	0.040
**MTHFR genotype**				
CC	Ref		Ref	
CT	1.787 (1.089, 2.934)	0.022	1.543 (0.873, 2.729)	0.136
TT	5.077 (2.598, 9.923)	<0.001	2.786 (1.233, 6.295)	0.014
*p* for trend		<0.001		0.039
**MRI features**				
**WMH**				
0	Ref		Ref	
1	2.275 (1.282, 4.035)	0.005	1.843 (0.936, 3.631)	0.077
2	3.103 (1.669, 5.771)	<0.001	2.518 (1.208, 5.250)	0.014
3	6.738 (3.070, 14.786)	<0.001	3.067 (1.225, 7.680)	0.017
*p* for trend		<0.001		0.41
CMB	0.693 (0.426, 1.130)	0.142		
Lacunes	1.957 (1.169, 3.276)	0.011	2.875 (1.518, 5.445)	0.001

CI, confidence interval; OR, odds ratio; HHcy, hyperhomocysteinemia; CC, CC genotype; CT, CT genotype; TT, TT genotype; WMH, white matter hyperintensity; CMB, cerebral microbleeds; *p* ≤ 0.05 was considered statistically significant.

## Discussion

CSVD represented a a progressively accumulating chronic injury process, which gradually affected patients’ cognitive abilities, emotional states, and capabilities for daily living. This imposed substantial economic and social burdens on both the patients themselves and their families (Markus and Erik de Leeuw, 2023). In this cross-sectional study, we investigated potential associations between the *MTHFR* C677T polymorphism and cognitive function. Our findings indicated that the *MTHFR* 677 TT genotype independently conferred increased risk for both progression of WMH and cognitive impairment in CSVD patients.

Although WMH was the most common radiological manifestation of CSVD, its insidious symptom onset often led to underestimation of true prevalence. A prior study based on a Chinese population revealed that the prevalence of periventricular hyperintensity (PVH) was 72.1%, and deep white matter hyperintensity (DWMH) prevalence of 65.4% among CSVD patients (Yang et al., 2023). In our study, the proportion of participants with WMH was 71.5%, consistent with these previous findings. While variations existed across races and WMH definitions among research reports, a notably high WMH prevalence persisted (Yang et al., 2023). We observed a marked increase in cognitive impairment prevalence within the WMH subgroup, emphasizing the association between WMH and cognitive decline.

The linkage between the *MTHFR* gene and elevated homocysteine levels has been widely acknowledged, and the relationship between genetically-mediated HHcy and cerebrovascular diseases represented a major focus of current clinical research (Li et al., 2022). The *MTHFR* C677T mutation included three polymorphisms (wild-type CC, heterozygous CT, and homozygous TT). The CC genotype exhibited the highest frequency (45.2%) while the TT genotype was least frequent (20.9%). Patients with the TT genotype exhibited significantly higher plasma homocysteine compared to those with CC and CT genotypes (*p* < 0.001). Elevated homocysteine may promote inflammatory responses via increased pro-inflammatory and reduced anti-inflammatory cytokines, consequently impairing vascular endothelial structure and function, enhancing vascular permeability, disrupting the blood–brain barrier, and ultimately resulting in ischemic and hypoxic injury to both white and gray matter (Al Mutairi, 2020). For equivalent damage, the homocysteine concentration required for cerebral small vessel endothelial injury was lower than for large vessels, indicating greater small vessel sensitivity to homocysteine. This further confirmed more pronounced damage to small vessels from elevated homocysteine (Feng et al., 2013). Our findings corroborate this perspective, patients possessing the TT genotype displayed heightened severity of WMH pathology and cognitive impairment compared to those harboring the CC and CT genotypes. However, we observed no statistically significant variations between genotypic groups regarding CMB and lacunar infarcts.

We further investigated factors associated with WMH, finding a significant positive correlation between the severity of white matter lesion severity and age, hypertension, homocysteine and *MTHFR* genotype (*p* < 0.001). Our results were consistent with other cohorts in showing that age and hypertension were independent risk factors for WMH (Yang et al., 2023). Hypertension can directly impair small arteries, precipitating atherosclerotic evolution, reflecting similar microvascular pathologies with CSVD. Concomitantly, it can induce lipoidosis, gradually compromising cerebral metabolism and elevating risk of cognitive impairment, with these consequences intensifying in conjunction with aging. The association between *MTHFR* C677T polymorphism and CSVD remained a topic of ongoing debate. Previous studies indicated robust associations between *MTHFR* C677T polymorphism and WMH volume (Rutten-Jacobs et al., 2016), and HHcy had been identified as a predictive factor for the severity of white matter lesions in elderly patients (Yu et al., 2020). However, Jeon et al. reported that despite the linkage between HHcy and small vessel disease (SVD), and the role of TT genotype as an important determinant of HHcy, the *MTHFR* C677T polymorphism was not related to SVD (Jeon et al., 2014). In this study, we observed age, hypertension, HHcy, TT genotype to be independent risk factors of WMH by multivariable logistic regression analysis. However, the association between the *MTHFR* C677T polymorphism and neuroimaging phenotypes of CSVD remains to be fully elucidated.

One previous study reported that WMH may increase the risks of vascular dementia by 73%, and consistently increased volume or severity of WMH shown to heighten dementia susceptibility (Hu et al., 2021). Remaining consistent with the perspective expressed above, our results demonstrated that CSVD patients with more severe WMH have a significantly increased risk of cognitive impairment. WMH may engender cognitive dysfunction through secondary demyelination and consequent neuronal loss (Wang et al., 2021). WMH were regarded as a crucial neuroimaging biomarker of cognitive impairment, and a more accurate assessment could be achieved through the measurement of WMH volume (Prins and Scheltens, 2015). Our study revealed a modest correlation between lacunar infarcts and cognitive impairment. Research shown approximately 40% of lacunar infarcts patients exhibit varying degrees of vascular cognitive impairment (Ohlmeier et al., 2023). WMH and lacunar infarcts may exhibit synergistic effects on cognitive impairment; therefore, future investigations focused on elucidating the intricate interrelationships between neuroimaging biomarkers and cognitive function in CSVD are imperative.

Although HHcy has been identified as a potential risk factor for dementia (Miwa et al., 2016; Teng et al., 2022), homocysteine levels were influenced by a multitude of both external and internal factors, hence, the impact of homocysteine on cardiovascular disease may exhibit a diverse effects. Our study revealed that more pronounced cognitive impairment in individuals with HHcy and the *MTHFR* 677 TT genotype. This implies that CSVD patients may develop more substantial cognitive impairment under specific genetic backgrounds. Given the positive correlation between WMH burden and risk of cognitive impairment, we can speculate that *MTHFR* 677 TT genotype may aggravate WMH by elevating serum homocysteine levels, thereby heightening susceptibility to cognitive decline. However, as a cross-sectional study, we could not establish definitive causal relationships between the MTHFR 677 TT genotype, WMH, and cognitive dysfunction. Future longitudinal cohort studies will help elucidate the interrelationships among these factors.

Cognitive impairment resulted from the complex interplay of multiple factors. Apart from the MTHFR 677 TT genotype as an important genetic risk factor, other hazards warranted attention as well. For instance, hypertension could engender atherosclerosis, while aging exacerbated β-amyloid deposition—both processes were implicated in cognitive decline. Although incorporating common risk factors like age, sex, smoking, hypertension, dyslipidemia, and diabetes, our study provides limited assessments of the complex multifactorial etiology underlying cognitive impairment. Further large-scale multifactor investigations will be warranted to construct sophisticated predictive models that appraise the impacts and interactions of diverse risk elements on cognitive dysfunction, vital steps for forecasting and preventing neurological morbidity. Although non-genetic or mixed factors may have played essential roles in the pathogenesis of homocysteine-induced vascular changes, our research advised that high-risk individuals with HHcy undergo screening for associated genetic risks, so preemptive interventions can mitigate cognitive impairment risk.

This study has several limitations. Firstly, the cross-sectional design of our study, is unable to investigate causality. Secondly, the single-center study with a relatively limited sample size, which may introduce selection bias and constrain generalizability beyond the sampled population. Due to the limited sample size, conducting risk factor analyses for lacunar infarctions and cerebral microbleeds was not feasible. Thus, larger multi-center studies are needed to enhance generalizability. Moreover, cognitive impairment typically arises from multifactorial influences, yet this study exclusively focuses on *MTHFR* gene variations, while interactions with other risk factors or potential confounders require further investigation. Future investigations should implement a prospective cohort design, augment sample sizes, and evaluate the comprehensive effects of diverse risk determinants to bolster the credibility of study conclusions.

## Conclusion

Through analyzing imaging characteristics, *MTHFR* C677T polymorphisms, and cognitive function in 330 CSVD patients, this study demonstrates the *MTHFR* 677 TT genotype as an independent genetic risk factor contributing to WMH and cognitive impairment in CSVD patients. These findings underscore the complex genetic underpinnings of CSVD, which may ultimately inform development of personalized preventative and therapeutic modalities for this condition. However, the cross-sectional design and limited sample size constrain generalizability and causal inferences, necessitating future large-scale, longitudinal investigations with a prospective cohort design to unravel the intricate pathogenic mechanisms underlying cognitive impairment in CSVD.

## Data availability statement

The original contributions presented in the study are publicly available. This data can be found here: China National GeneBank Database, CNP0005057.

## Ethics statement

The studies involving humans were approved by the Affiliated Mindong Hospital of Fujian Medical University. The studies were conducted in accordance with the local legislation and institutional requirements. The participants provided their written informed consent to participate in this study.

## Author contributions

QW: Investigation, Methodology, Writing – original draft. CY: Formal analysis, Investigation, Methodology, Writing – original draft. ZZ: Conceptualization, Data curation, Supervision, Writing – review & editing. CC: Data curation, Investigation, Project administration, Supervision, Writing – review & editing. XZ: Data curation, Formal analysis, Investigation, Writing – original draft. XL: Data curation, Formal analysis, Funding acquisition, Investigation, Project administration, Writing – original draft.

## References

[ref1] Al Mutairi F. (2020). Hyperhomocysteinemia: clinical insights. J. Cent. Nerv. Syst. Dis. 12:1179573520962230. doi: 10.1177/1179573520962230, PMID: 33100834 PMC7549175

[ref2] Creavin S. T. Wisniewski S. Noel-Storr A. H. Trevelyan C. M. Hampton T. Rayment D.. (2016). Mini-mental state examination (MMSE) for the detection of dementia in clinically unevaluated people aged 65 and over in community and primary care populations. Cochrane Database Syst. Rev. 2016:CD011145. doi: 10.1002/14651858.CD011145.pub2, PMID: 26760674 PMC8812342

[ref3] Feng C. Bai X. Xu Y. Hua T. Huang J. Liu X.-Y. (2013). Hyperhomocysteinemia associates with small vessel disease more closely than large vessel disease. Int. J. Med. Sci. 10, 408–412. doi: 10.7150/ijms.5272, PMID: 23471237 PMC3590600

[ref4] Hassan A. Hunt B. J. O’Sullivan M. Bell R. D’Souza R. Jeffery S.. (2004). Homocysteine is a risk factor for cerebral small vessel disease, acting via endothelial dysfunction. Brain J. Neurol. 127, 212–219. doi: 10.1093/brain/awh023, PMID: 14607791

[ref5] Hu H.-Y. Ou Y.-N. Shen X.-N. Qu Y. Ma Y.-H. Wang Z.-T.. (2021). White matter hyperintensities and risks of cognitive impairment and dementia: a systematic review and meta-analysis of 36 prospective studies. Neurosci. Biobehav. Rev. 120, 16–27. doi: 10.1016/j.neubiorev.2020.11.007, PMID: 33188821

[ref6] Jeon S.-B. Kang D.-W. Kim J. S. Kwon S. U. (2014). Homocysteine, small-vessel disease, and atherosclerosis: an MRI study of 825 stroke patients. Neurology 83, 695–701. doi: 10.1212/WNL.0000000000000720, PMID: 25031284

[ref7] Ji Y. Li X. Teng Z. Li X. Jin W. Lv P. Y. (2020). Homocysteine is associated with the development of cerebral small vessel disease: retrospective analyses from neuroimaging and cognitive outcomes. J. Stroke Cerebrovasc. Dis. Off. J. Natl. Stroke Assoc. 29:105393. doi: 10.1016/j.jstrokecerebrovasdis.2020.105393 33254368

[ref8] Li Z. Wu X. Huang H. Xu F. Liang G. Lin C.. (2022). MTHFR C677T polymorphism and cerebrovascular lesions in elderly patients with CSVD: a correlation analysis. Front. Genet. 13:987519. doi: 10.3389/fgene.2022.987519, PMID: 36212120 PMC9537945

[ref9] Liu S. Sun Z. Chu P. Li H. Ahsan A. Zhou Z.. (2017). EGCG protects against homocysteine-induced human umbilical vein endothelial cells apoptosis by modulating mitochondrial-dependent apoptotic signaling and PI3K/Akt/eNOS signaling pathways. Apoptosis Int. J. Program. Cell Death 22, 672–680. doi: 10.1007/s10495-017-1360-8, PMID: 28317089

[ref10] Markus H. S. Erik de Leeuw F. (2023). Cerebral small vessel disease: recent advances and future directions. Int. J. Stroke 18, 4–14. doi: 10.1177/17474930221144911, PMID: 36575578 PMC9806465

[ref11] Miwa K. Tanaka M. Okazaki S. Yagita Y. Sakaguchi M. Mochizuki H.. (2016). Increased Total homocysteine levels predict the risk of incident dementia independent of cerebral small-vessel diseases and vascular risk factors. J. Alzheimers Dis. 49, 503–513. doi: 10.3233/JAD-150458, PMID: 26484913

[ref12] Ohlmeier L. Nannoni S. Pallucca C. Brown R. B. Loubiere L. Markus H. S.. (2023). Prevalence of, and risk factors for, cognitive impairment in lacunar stroke. Int. J. Stroke Off. J. Int. Stroke Soc. 18, 62–69. doi: 10.1177/17474930211064965, PMID: 34983273 PMC9806466

[ref13] Prins N. D. Scheltens P. (2015). White matter hyperintensities, cognitive impairment and dementia: an update. Nat. Rev. Neurol. 11, 157–165. doi: 10.1038/nrneurol.2015.10, PMID: 25686760

[ref14] Rutten-Jacobs L. C. Traylor M. Adib-Samii P. Thijs V. Sudlow C. Rothwell P. M.. (2016). Association of MTHFR C677T genotype with ischemic stroke is confined to cerebral small vessel disease subtype. Stroke 47, 646–651. doi: 10.1161/STROKEAHA.115.011545, PMID: 26839351 PMC4760380

[ref15] Teng Z. Feng J. Liu R. Ji Y. Xu J. Jiang X.. (2022). Cerebral small vessel disease mediates the association between homocysteine and cognitive function. Front. Aging Neurosci. 14:868777. doi: 10.3389/fnagi.2022.868777, PMID: 35912072 PMC9335204

[ref16] Wang T. Jin A. Fu Y. Zhang Z. Li S. Wang D.. (2021). Heterogeneity of white matter Hyperintensities in cognitively impaired patients with cerebral small vessel disease. Front. Immunol. 12:803504. doi: 10.3389/fimmu.2021.803504, PMID: 34956241 PMC8695488

[ref17] Wardlaw J. M. Smith E. E. Biessels G. J. Cordonnier C. Fazekas F. Frayne R.. (2013). Neuroimaging standards for research into small vessel disease and its contribution to ageing and neurodegeneration. Lancet Neurol. 12, 822–838. doi: 10.1016/S1474-4422(13)70124-8, PMID: 23867200 PMC3714437

[ref18] Wardlaw J. M. Smith C. Dichgans M. (2019). Small vessel disease: mechanisms and clinical implications. Lancet Neurol. 18, 684–696. doi: 10.1016/S1474-4422(19)30079-1 31097385

[ref19] Yang Y. Cai X. Zhou M. Chen Y. Pi J. Zhao M.. (2023). Prevalence and risk factors of cerebral small vessel disease from a population-based cohort in China. Neuroepidemiology 57, 413–422. doi: 10.1159/000533678, PMID: 37734325

[ref20] Yu L. Yang L. Li Y. Yang S. Gu H. Hu W.. (2020). Hyperhomocysteinemia can predict the severity of white matter hyperintensities in elderly lacunar infarction patients. Int. J. Neurosci. 130, 231–236. doi: 10.1080/00207454.2019.1667795, PMID: 31744348

